# Fibroblast growth factor 21 response to critical illness: effect of blood glucose control and relation with mitochondrial dysfunction, the integrated stress response and survival

**DOI:** 10.1186/2197-425X-3-S1-A977

**Published:** 2015-10-01

**Authors:** S Thiessen, I Vanhorebeek, I Derese, J Gunst, G Van den Berghe

**Affiliations:** KU Leuven, Clinical Division and Laboratory of Intensive Care Medicine, Department of Cellular and Molecular Medicine, Leuven, Belgium

## Introduction

Fibroblast growth factor 21 (FGF21) is a recently identified hormone that regulates metabolic homeostasis, with a potential role as a stress hormone [1]. Critical illness is hallmarked by insulin resistance and mitochondrial damage, metabolic alterations that increase circulating levels of FGF21 via the integrated stress response (ISR) [2]. Targeting normoglycemia during critical illness has shown to attenuate mitochondrial damage in the liver, the main source of FGF21 [3].

## Objectives

We hypothesized that critical illness elevates FGF21 serum concentrations, possibly induced by mitochondrial damage and brought about by ISR mediators. We also hypothesized that targeting normoglycemia during critical illness suppresses FGF21 serum concentrations and *fgf21* liver expression, possibly by diminishing mitochondrial damage in vital organs.

## Methods

FGF21 serum concentrations were quantified in 405 fed patients requiring intensive care for at least 7 days, who were randomized to normoglycemia or tolerating hyperglycemia, and in 20 matched controls. In 39 rabbits, of which 26 were critically ill and randomly assigned to normoglycemia or hyperglycemia and 13 were healthy controls, *fgf21* expression was quantified on day 3 or day 7 in liver, kidney, muscle and adipose tissue. Correlations between *fgf21* expression and mitochondrial respiratory chain enzyme complex activities and ISR mediators were analyzed.

## Results

In the critically ill patients, serum FGF21 concentrations upon ICU admission were 8-fold higher than those in 20 healthy matched control subjects (p < 0.0001), decreased with time, but always remained higher in non-survivors than in survivors (p≤0.006). Maintaining normoglycemia lowered serum FGF21 (p = 0.01), statistically explaining at least part of its mortality benefit. Hepatic *fgf21* expression in critically ill rabbits was substantially increased on day 3 and day 7 (p≤0.01) and tightly correlated with mitochondrial dysfunction (all R^2^≥0.49, all p≤0.0006 for complex I and V) on both days and with markers of the ISR on day 3 (R^2^≥0.73, p≤0.0001), which were all lowered by targeting normoglycemia. In kidney, muscle and adipose tissue, *fgf21* expression was undetectable.

## Conclusions

Critical illness was identified as a potent inducer of serum FGF21 concentrations and liver *fgf21* expression, possibly driven at least in part by mitochondrial damage and the ISR, which were all attenuated by targeting normoglycemia.

## Grant Acknowledgment

S. Thiessen received a FWO Research Assistant Fellowship (Aspirant). G. Van den Berghe receives long-term structural research financing via the Methusalem program, funded by the Flemish government (METH/08/07) and holds a European Research Council Advanced Grant (AdvG-2012-321670) from the Ideas Programme of the EU FP7.

## References

[1] Itoh (2014). Front Endocrinol.

[2] Kim (2013). Nat Med.

[3] Vanhorebeek (2005). Lancet.

